# The marginality principle revisited: Should “higher‐order” terms always be accompanied by “lower‐order” terms in regression analyses?

**DOI:** 10.1002/bimj.202300069

**Published:** 2023-09-29

**Authors:** Tim P. Morris, Maarten van Smeden, Tra My Pham

**Affiliations:** ^1^ MRC Clinical Trials Unit at UCL London UK; ^2^ Julius Center for Health Sciences and Primary Care, University Medical Center Utrecht University Utrecht The Netherlands

**Keywords:** curse of dimensionality, interactions, marginality principle, polynomials, principle of marginality, ratios

## Abstract

The *marginality principle* guides analysts to avoid omitting lower‐order terms from models in which higher‐order terms are included as covariates. Lower‐order terms are viewed as “marginal” to higher‐order terms. We consider how this principle applies to three cases: regression models that may include the ratio of two measured variables; polynomial transformations of a measured variable; and factorial arrangements of defined interventions. For each case, we show that which terms or transformations are considered to be lower‐order, and therefore marginal, depends on the scale of measurement, which is frequently arbitrary. Understanding the implications of this point leads to an intuitive understanding of the curse of dimensionality. We conclude that the marginality principle may be useful to analysts in some specific cases but caution against invoking it as a context‐free recipe.

## 1 INTRODUCTION

Regression models frequently include covariates that were not directly measured but were calculated as functions of the directly‐measured variables. An obvious example is an interaction variable, which is computed as the product of two or more measured variables. Further examples include ratios, polynomials, and other nonlinear transformations of the directly measured variables. All these cases involve some manipulation of the measured variables.

A principle sometimes referred to as the *marginality principle* aims to guide how analysts should include such terms in regression models. The idea is that higher‐order terms (e.g., an interaction, a ratio, or a polynomial) should only appear in a regression model when accompanied by lower‐order terms. We associate the term *marginality* with Nelder (1977), who expressed dissatisfaction with “The Neglect of Marginality.” Nelder was interested in factorial experiments and described models which neglect marginality, by including interactions without their main effects, as “of no practical interest.” While interactions in factorial experiments seems to be the context in which the principle was first discussed, the idea extends naturally to other contexts, and certainly had been by 1989:

As elsewhere, it is important that the final model or models should make sense physically: at a minimum, this usually means that interactions should not be included without their main effects not higher‐degree polynomial terms without their lower‐degree relatives.

– (McCullagh & Nelder, 1989, Section 3.9, p. 89)

This article claims that the marginality principle is far more subtle than it may appear. In particular, we show that the idea of certain variables being “marginal” to others is often arbitrary. The article is structured around three cases: ratios, polynomials, and factorial experiments. For each, we show that violating the principle can make sense. There are close links between the three cases but we regard it as helpful to view the arguments from these three angles. The principle is widespread and these three cases include issues relate to different aims, spanning *design*, *pre‐specified analysis* and *model selection*.

## 2 CASE 1: RATIO VARIABLES (AND INTERACTIONS)

In health research, the use of body mass index (BMI), the ratio of a person's weight in kilograms to squared‐height (in metres), is widespread. Let *A* and *B* denote two measured variables and R=A/B their ratio. In this case, *R* is typically viewed as “higher‐order.” Some other examples of ratios include waist‐to‐hip ratio (Carlsson et al., 2020), total cholesterol to low‐density lipoprotein (Arsenault et al., 2009), the ratio of urinary albumin to creatinine (Heerspink et al., 2020), left ventricular ejection fraction (stroke volume divided by end‐diastolic volume) (Fabijanovic et al., 2019), and resting heart rate (beats‐per‐minute, BPM) (Seviiri et al., 2017). Further examples can be found in Kronmal (1993).

There are various papers in the literature that argue against the use of ratio variables as covariates in regression models; see, for example, Westfall (2015), or Wiseman (2009) and references therein. The argument tends to follow the line that it is not proper to include *R* without including *A* and *B*; that it would be more appropriate to include *A* and *B* separately and omit *R*. This view is clearly informed by the marginality principle.

Consider the ratio BPM as a measurement of heart rate. As its name implies, BPM is a ratio variable calculated by dividing “number of beats counted” by “measurement time (in minutes).” Mayo Clinic recommends recording the number of beats over a period of 15 seconds and multiplying the count by four (Laskowski, 2020). Suppose that, in a given data set, all participants' heart rates are measured over 15 seconds. It is likely that an analyst would be happy to include only BPM, and not the number of counted beats, as a covariate in a regression model, since this only changes the scale of the coefficient in the model. Furthermore, including the denominator variable *A*—which is constant at 15 seconds—would lead to an inestimable parameter in a regression model (provided the model includes an intercept). In this example, it makes no sense to follow the marginality principle.

In contrast to the Mayo clinic, the National Health Service (NHS) website advises counting the number of beats over 30 or 60 s (NHS, 2021) (for the purposes of this article we will ignore measurement error but note that its magnitude will likely be affected by the length of recording time).

Suppose a data set contains data from individuals where the number of beats recorded may have followed one of the three possible procedures described on the Mayo Clinic and NHS websites, and so may be counted over 15, 30, or 60 seconds (Laskowski, 2020; NHS, 2021). The number of minutes *B* is no longer constant across individuals. It could be included as a covariate in a regression model with or without the number of beats *A* or BPM *R*. This would again make no sense: the length of time for which beats were recorded does not represent any measurement about the individual but a choice of the data collector. It is unlikely that an analyst would argue to include beats *A* and minutes *B* instead of the ratio BPM *R*. BPM is an uncontroversial way of standardising measurements of *A* according to *B* and so we believe most analysts would again agree that it makes practical sense to ignore the marginality principle.

The simple example of BPM emphasises two points. First, that there is nothing inherently wrong with including a ratio without its components in a regression model, which is commonly done and commonly criticised. Second, in this particular example it is possible to directly measure any of *A*, *B*, and *R*. “Ratios” are labelled as such because they are formed by deriving *R* from measured variables *A* and *B*. This is not always the case: a car's speedometer shows the velocity (a ratio) but not distance or time.

Rather than directly measuring *A* and *B* to derive *R*, suppose that we measured variables *A* and *R*, but not *B*. We could then derive *B* as A/R. Notice that *B* is the ratio of the two measured variables, *A* and *R*. Any analyst who above objects to a regression model that includes *R* without *A* and/or *B* will now object to a model that includes *B*—a ratio—without *A* and/or *R*. Is it still better to include (A,B) alone, rather than *R* alone, (A,R) or (B,R)?

Finally, suppose that we directly measure *R* and *B* but not *A*; we can then derive A=B×R, which is a product, much like an interaction. Any analyst who initially wished to include *A* as a main effect without *R* must surely now object. *A* is not a main effect but the interaction of *B* with *R*, and so is the higher‐order term. Including it as a covariate in the absence of *B* and *R* violates the marginality principle.

To summarize, the notion of *marginality* seems to take the function as inherently defined R:=A/B where it is more appropriate to regard it as simply the function that happened to be calculated R=A/B, which can be rearranged as A=R/B or B=A/R. The regression model

(1) $$Y_{i}=\beta_{0}+\beta_{1}A_{i}+\beta_{2}B_{i}+\beta_{3}R_{i}+\varepsilon_{i}$$

may be rewritten as

(2) $$Y_{i}=\beta_{0}+\beta_{1}\left(B_{i}R_{i}\right)+\beta_{2}\frac{A_{i}}{R_{i}}+\beta_{3}\frac{A_{i}}{B_{i}}+\varepsilon_{i}.$$

Here, we have denoted the covariates as higher‐order terms, but their values in any data set are identical and so models (1) and (2) are identical. The same model may be written in any of the following ways:

$$\begin{array}{cc} Y_{i}=\; & \beta_{0}+\beta_{1}A_{i}+\beta_{2}B_{i}+\beta_{3}\frac{A_{i}}{B_{i}}+\varepsilon_{i}\\ Y_{i}=\; & \beta_{0}+\beta_{1}A_{i}+\beta_{2}\frac{A_{i}}{R_{i}}+\beta_{3}R_{i}+\varepsilon_{i}\\ Y_{i}=\; & \beta_{0}+\beta_{1}\left(B_{i}R_{i}\right)+\beta_{2}B_{i}+\beta_{3}R_{i}+\varepsilon_{i}\\ Y_{i}=\; & \beta_{0}+\beta_{1}A_{i}+\beta_{2}\frac{A_{i}}{R_{i}}+\beta_{3}\frac{A_{i}}{B_{i}}+\varepsilon_{i}\\ Y_{i}=\; & \beta_{0}+\beta_{1}\left(B_{i}R_{i}\right)+\beta_{2}B_{i}+\beta_{3}\frac{A_{i}}{B_{i}}+\varepsilon_{i}\\ Y_{i}=\; & \beta_{0}+\beta_{1}\left(B_{i}R_{i}\right)+\beta_{2}\frac{A_{i}}{R_{i}}+\beta_{3}R_{i}+\varepsilon_{i}. \end{array}$$

## 3 CASE 2: POLYNOMIAL TRANSFORMATIONS

Venables (2000) discusses polynomials in similar terms to interactions. A first‐order interaction is calculated as the product of two variables. The square of a continuous variable is the product of that variable with itself, and so may be viewed as analogous to a first‐order interaction, the cube as analogous to a second‐order interaction, and so on.

**FIGURE 1:**
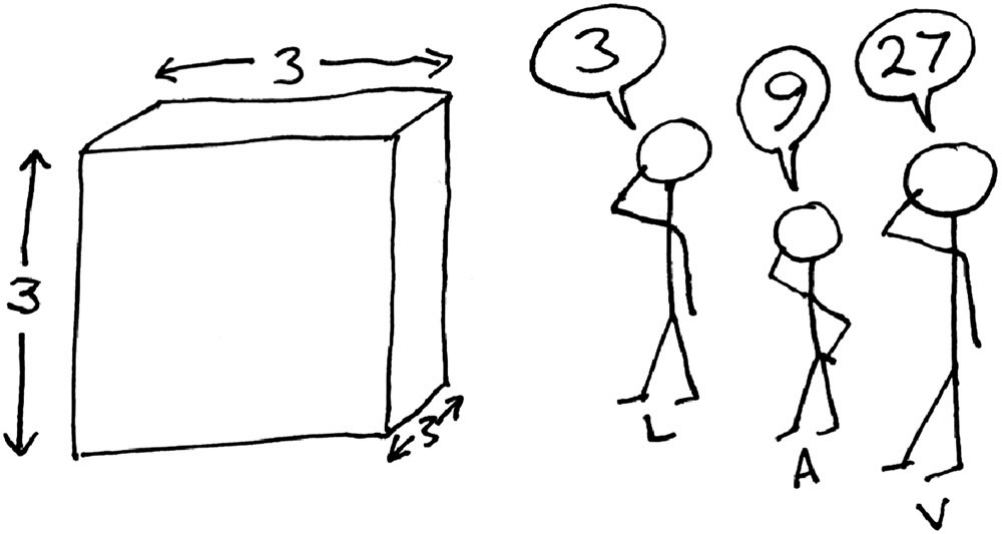
Three scientists are asked, “What is the size of this object?”

Is it obvious in the case of polynomials which transformations are lower‐ and higher‐order? No. To see why, consider the following example. Three scientists, named *L*, *A*, and *V* are asked to measure the size of an object (which is a cube). Figure 1 shows the cube and the scientists' responses: scientist *L* answers “3,” scientist *A* answers “9,” and scientist *V* answers “27.” Clearly *L* has measured the Length of one side, *A* has measured the Area of one face, and *V* has measured the Volume of the cube. Each scientist is perplexed by the others' responses. *L* thinks *A* has squared the size and *V* has cubed it. *A* thinks *L* has taken the square root of the size and *V* has raised it to the power 1.5. *V* thinks *L* has measured the cube‐root and *A* the 2/3‐root.

Who has provided the correct, lower‐order measurement of this object and who has provided a transformed, higher‐order measurement? Readers who think there is a clear answer may reconsider when told the relevance to each scientist:

- *L* needs to stand on top of it to reach something;
- *A* has been asked to paint the top;
- *V* wants to fill it with liquid.

The example again demonstrates that the designation of “lower‐” and “higher‐” order terms is arbitrary and depends on how we measure something. As with Case 2, this makes it hard to know how to use the marginality principle.

## 4 CASE 3: FACTORIAL EXPERIMENTS

The previous examples focus on quantitative variables, but Nelder's initial focus was on factorial experiments (Nelder, 1977). In the discussion of Nelder (1977), Dennis Lindley posed a clever question:

I suppose that with the mud of Rothamsted on his boots, Dr Nelder is in a better position than an academic like me to comment on practical matters, but his assertion that an interaction with no main effects is of no practical interest seems rash. A main effect can be turned into an interaction and vice versa. Consider two factors each at levels 0 and 1 with yields as follows:

**Table:** 

	0	1
0	3	5
1	3	5

with one main effect and no interaction. Let one factor remain unaltered but let the other be at level 0 if the original two factors were at the same level, and 1 otherwise. The new factors have no main effects, only an interaction. The important point is what inferences are to be made?

Nelder's response was abrupt and dismissive (Nelder, 1977):

The process is analogous to the rotation of axes in multivariate methods. Like models which violate marginality relations, rotation procedures are well defined mathematically but may not make any practical sense.

Lindley's point was provocative, and analogous to our discussion of ratios and polynomials. The mathematical point was that, given two treatments, each with two levels, we can encode the levels as 1 and 2 (rather than 0 and 1) and the original two treatments are ratios (treatment *A* can be computed from the interaction AB divided by *B*). The relevance to the marginality principle is that what we regard as marginal depends on the encoding—or measurement—used. This argument extends naturally to factorial designs with more than two factors.

The following example relates to Lindley's point. Suppose a new surgical procedure is developed that surgeons require training to perform. One obvious approach to evaluating this intervention would be to run a two‐arm trial, randomising $n_{soc}$ participants to standard‐of‐care and $n_{new}$ to new‐procedure‐conducted‐by‐a‐trained‐surgeon. Suppose a critic were to argue that such a trial entangles the effects of two factors: the surgeon's training and the procedure itself. They explain the problem and solution using the following tables:

**Table:** 

Two arms		
	*A*	$A^{\prime }$
*B*	$n_{new}$	0
$B^{\prime }$	0	$n_{soc}$

**Table:** 

2 × 2 factorial		
	*A*	$A^{\prime }$
*B*	$n_{A,B}$	$n_{A^{\prime },B}$
$B^{\prime }$	$n_{A,B^{\prime }}$	$n_{A^{\prime },B^{\prime }}$

The procedure is not a single intervention but has two components: *new versus old intervention* and *surgeon versus non‐surgeon*. Thus, a factorial trial should be designed to understand not just whether to use this procedure but to understand the contribution of its two components and whether they are synergistic or antagonistic when combined. It is clear that the interaction—the new procedure delivered by a trained surgeon—is of substantive interest. The effect of either component in the absence of the other is of no interest and so the suggestion to study the question using a 2 × 2 factorial design is absurd. That is, respecting marginality is nonsensical in this context.

## 5 DISCUSSION

The marginality principle is stated on Wikipedia as follows (Wikipedia, 2022):

1. In a regression model including an interaction term between two variables, it is wrong to omit their main effects;
2. When an interaction exists, it is wrong to include the variables' main effects from a regression model and omit their interaction.

The presence of the second point is curious, since at the date of writing it is not justified or explained by any of the references therein. Most analysts are taught to consider only the first aspect. In our BPM example, it was uncontroversial to say that BPM measures something of medical interest not captured by beats or by minutes alone. The situation is far less clear for BMI. The usual advice from statistical critics of BMI seems to be that weight and height should be included in the model separately. This seems to invoke the marginality principle (include the marginal effects of weight and height before considering measure of weight standardised for height). Of course, this does not fully respect the principle. Further, if there is substantive interest in BMI as defined, excluding it would not be of interest.

Recent work by Berrie et al. (2022) considers how to represent and use derived variables on a causal DAG (directed acyclic graph), which has some surprising links to the marginality principle. They argue that components cannot simply be included in causal DAGs as though they were probabilistic causes of the whole derived variable. Instead, they view components as *being* the whole. Their proposal is then to depict deterministic derived variables as a node taking directed edges with a double‐lined arrow from the components. This representation, and identification logic that follows, encodes assumptions in a very similar way to the marginality principle. We regard the choice to make the double‐lined edges directional as mistaken: it implies that there is something more “concrete” about the components than the derived variable. The examples in the present article demonstrate that this is often not true. Their strategy results in the argument that derived variables, such as BMI, should often be excluded from analyses. This contradicts the premise of components *being* the whole, and leads the researcher reading the causal DAG to act as though derived variables are standard probabilistic causes of the derived variable.

In the absence of prior knowledge, we cannot say which variables, *A*, *B*, or *R* we need to include as covariates in a regression model. Simply mimicking other analysts' procedures may perpetuate their mistakes.

Making decisions to omit higher‐order terms from regression models may often be done because such terms are hard to conceptualize. However, we can easily make qualitative statements about higher‐order interactions in everyday life. We may feel confident to say “My skin will get wet if it is raining and I go outside unclothed but it will stay dry if it is not raining, if I stay inside, I wear waterproof clothing or if use an umbrella” or “Adding either chocolate sauce or hot sauce improves the taste of some food but adding both never does.” When faced with data to analyse, we typically have very little knowledge of the generative mechanism, meaning that we may unwittingly fit models analogous to “Adding chocolate sauce improves some food and adding hot sauce improves some food” without the qualifier about not adding both (the interaction), or “One's skin gets wet if it is raining or if one is unclothed or if one goes outside.”

We have questioned the sense of blindly following the marginality principle. However, our arguments are based on measurement of covariates. The marginality principle is also used to justify including an intercept term in regression models, and this is much more fundamental. Consider temperature, which may be measured in Celsius or Fahrenheit. We can convert between them using 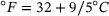 or 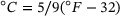. Including temperature as a covariate in a model but excluding the intercept term means that inference depends on the measurement scale.

We have considered single‐level models in an attempt to distil the problem. In the context of multi‐centre trials, Senn (2000) used the marginality principle as one of several arguments against type III sums of squares. This is, in our view, a sensible use of the principle, since this case is much closer to the case of intercept terms than to arbitrary measurement of covariate values.

So what is the solution for the analyst? For a ratio variable, a simple approach, which superficially appears to fully respect the principle, would be to include all three variables, *A*, *B*, and *R* as covariates in a model. However, considering our arguments about polynomial transformations of continuous variables, we see that the choice of scale for these variables is also arbitrary: why was *A* measured rather than $\sqrt{A}$, *A* ^2^, or any other function? Once we understand the marginality principle, aiming to adhere to it leads us straight to the *curse of dimensionality* (Hernan & Robins, 2022, for example). We must make decisions about which terms to include and which to omit on some basis other than the marginality principle. Fractional polynomial models take the approach of admitting covariates up to a certain dimension (Royston & Sauerbrei, 2008) and, interestingly, do not attempt to respect the naive marginality; rather, the (restricted) set of candidate transformations of continuous covariates are treated as parameters.

The marginality principle provides an attractive context‐free recipe in which the directly‐measured variables automatically dominate functions derived from them. We have shown that this can lead to nonsensical and arbitrary choices about the form of covariates to include in a model, questioning the sense of the principle. Such choices cannot be reduced in this way and should be argued on a firmer footing.

## CONFLICT OF INTEREST STATEMENT

The authors declare no conflicts of interest.

## Data Availability

Not applicable; no data used for this article.
